# *N*-Methyl-2-(1-methyl-3-phenyl­prop-2-en-1-yl­idene)hydrazinecarbo­thio­amide

**DOI:** 10.1107/S1600536814013889

**Published:** 2014-06-21

**Authors:** Fillipe Vieira Rocha, Adelino Vieira de Godoy Netto, Johannes Beck, Jörg Daniels, Adriano Bof de Oliveira

**Affiliations:** a Instituto de Química, Universidade Estadual Paulista, Rua Francisco Degni s/n, 14801-970 Araraquara-SP, Brazil; b Institut für Anorganische Chemie, Universität Bonn, Gerhard-Domagk-Strasse 1, D-53121 Bonn, Germany; c Departamento de Química, Universidade Federal de Sergipe, Av. Marechal Rondon s/n, Campus, 49100-000 São Cristóvão-SE, Brazil

**Keywords:** crystal structure

## Abstract

In the title compound, C_12_H_15_N_3_S, the mol­ecule deviates slightly from planarity, with a maximum deviation from the mean plane of the non-H atoms of 0.2756 (6) Å for the S atom and a torsion angle for the N—N—C—N fragment of −7.04 (16)°. In the crystal, mol­ecules are linked by N—H⋯S hydrogen-bond inter­actions, forming centrosymmetric dimers. Additionally, one weak intra­molecular N—H⋯N hydrogen-bond inter­action is observed. The crystal packing shows a herringbone arrangement viewed along the *c* axis.

## Related literature   

For one of the first reports of the synthesis of thio­semicarbazone derivatives, see: Freund & Schander (1902 ▶). For a report of the anti­fungal activity of the title compound, see: Nishimura *et al.* (1979 ▶).
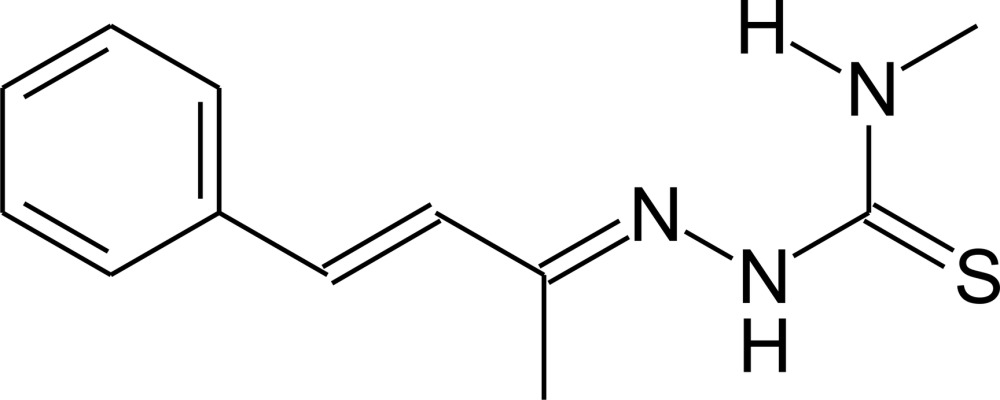


## Experimental   

### 

#### Crystal data  

C_12_H_15_N_3_S
*M* *_r_* = 233.33
Orthorhombic, 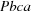

*a* = 10.5832 (2) Å
*b* = 7.9509 (2) Å
*c* = 28.9259 (5) Å
*V* = 2434.00 (9) Å^3^
*Z* = 8
Mo *K*α radiation
μ = 0.24 mm^−1^
*T* = 123 K
0.44 × 0.31 × 0.27 mm

#### Data collection  

Nonius KappaCCD diffractometer
Absorption correction: multi-scan (Blessing, 1995 ▶) *T*_min_ = 0.904, *T*_max_ = 0.955
26770 measured reflections
2783 independent reflections
2414 reflections with *I* > 2σ(*I*)
*R*_int_ = 0.046

#### Refinement  

*R*[*F* ^2^ > 2σ(*F* ^2^)] = 0.031
*wR*(*F* ^2^) = 0.080
*S* = 1.05
2783 reflections
205 parameters
All H-atom parameters refined
Δρ_max_ = 0.27 e Å^−3^
Δρ_min_ = −0.20 e Å^−3^

### 

Data collection: *COLLECT* (Nonius, 1998 ▶); cell refinement: *SCALEPACK* (Otwinowski & Minor, 1997 ▶); data reduction: *DENZO* (Otwinowski & Minor, 1997 ▶) and *SCALEPACK*; program(s) used to solve structure: *SHELXS97* (Sheldrick, 2008 ▶); program(s) used to refine structure: *SHELXL97* (Sheldrick, 2008 ▶); molecular graphics: *DIAMOND* (Brandenburg, 2006 ▶); software used to prepare material for publication: *publCIF* (Westrip, 2010 ▶).

## Supplementary Material

Crystal structure: contains datablock(s) I, publication_text. DOI: 10.1107/S1600536814013889/bx2460sup1.cif

Structure factors: contains datablock(s) I. DOI: 10.1107/S1600536814013889/bx2460Isup2.hkl

Supporting information file. DOI: 10.1107/S1600536814013889/bx2460Isup3.cml

CCDC reference: 1008277

Additional supporting information:  crystallographic information; 3D view; checkCIF report

## Figures and Tables

**Table 1:** Hydrogen-bond geometry (Å, °)

*D*—H⋯*A*	*D*—H	H⋯*A*	*D*⋯*A*	*D*—H⋯*A*
N3—H*N*3⋯N1	0.879 (17)	2.143 (16)	2.5877 (15)	110.7 (13)
N2—H*N*2⋯S1^i^	0.862 (18)	2.663 (18)	3.4296 (12)	148.7 (15)

Symmetry code: (i) 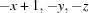.

**Fig. 1.**
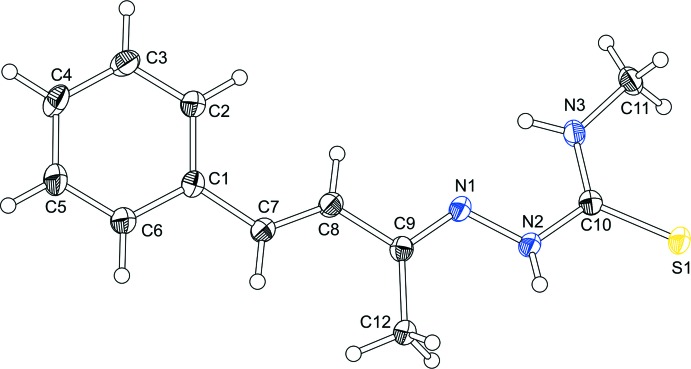
: The molecular structure of the title compound with labeling and displacement ellipsoids drawn at the 50% probability level.

**Fig. 2.**
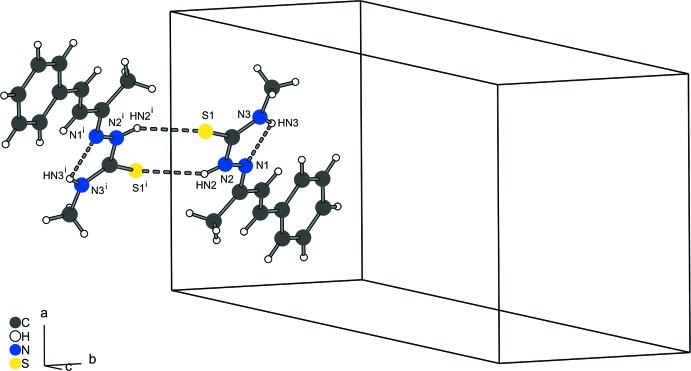
: Part of the crystal structure of the title compound showing the inter- and intramolecular hydrogen bonding as dashed lines. Symmetry code: (i) -x + 1, -y, -z.

**Fig. 3.**
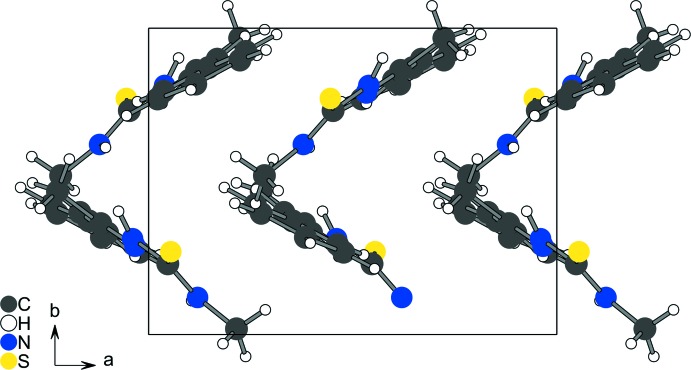
: Crystal structure of the title compound viewed along the c-axis. The herringbone pattern of the crystal packing along the a-axis is observed.

## supplementary crystallographic information

### S1. Comment

Thiosemicarbazone derivatives have a wide range of biological properties. For example, some thiosemicarbazones similar to the title compound show antifungal activity (Nishimura *et al.*, 1979). As part of our study on synthesis and structural chemistry of thiosemicarbazone derivatives from natural products, we report herein the crystal structure of a derivative of the essential oil of cinnamon bark (benzylideneacetone, a methyl derivative of the cinnamaldehyde).

In the crystal structure of the title compound the central N–N–C–N unit is not planar with an torsion angle along N1–N2–C10–N3 of -7.04 (16)° and the maximum deviation from the mean plane of the non-H atoms amounting to 0.2756 (6) Å for S1. The molecule, shows a *trans* conformation at the C7—C8 and N1—N2 bonds (Fig. 1).

In the crystal the molecules are linked by N—H···S hydrogen bonds interactions forming centrosymmetric dimers. Additionally, one weak N—H···N intramolecular H-interaction is observed.The crystal packing shows a herringbone arrangement viewed along the *c*-axis.(Fig. 3).

### S2. Experimental

Starting materials were commercially available and were used without further purification. The title compound synthesis was adapted to a procedure reported previously (Freund & Schander, 1902). The hydrochloric acid catalyzed reaction, a mixture of benzylideneacetone (10 mmol) and 4-methyl-3-thiosemicarbazide (10 mmol) in ethanol (80 ml) was refluxed for 5 h. After cooling and filtering, the title compound was obtained. Crystals suitable for X-ray diffraction were obtained in ethanol by the slow evaporation of solvent.

### S3. Refinement

All hydrogen atoms were localized in a difference density Fourier map. Their positions and isotropic displacement parameters were refined.

### Crystal data

**Table:**

C_12_H_15_N_3_S	*F*(000) = 992
*M**_r_* = 233.33	*D*_x_ = 1.273 Mg m^−^^3^
Orthorhombic, *P**b**c**a*	Mo *K*α radiation, λ = 0.71073 Å
Hall symbol: -P 2ac 2ab	Cell parameters from 31577 reflections
*a* = 10.5832 (2) Å	θ = 2.9–27.5°
*b* = 7.9509 (2) Å	µ = 0.24 mm^−^^1^
*c* = 28.9259 (5) Å	*T* = 123 K
*V* = 2434.00 (9) Å^3^	Fragment, yellow
*Z* = 8	0.44 × 0.31 × 0.27 mm

### Data collection

**Table:**

Nonius KappaCCD diffractometer	2783 independent reflections
Radiation source: fine-focus sealed tube, Nonius KappaCCD	2414 reflections with *I* > 2σ(*I*)
Graphite monochromator	*R*_int_ = 0.046
Detector resolution: 9 pixels mm^-1^	θ_max_ = 27.5°, θ_min_ = 3.3°
CCD rotation images, thick slices scans	*h* = −13→13
Absorption correction: multi-scan (Blessing, 1995)	*k* = −10→10
*T*_min_ = 0.904, *T*_max_ = 0.955	*l* = −37→37
26770 measured reflections	

### Refinement

**Table:**

Refinement on *F*^2^	Primary atom site location: structure-invariant direct methods
Least-squares matrix: full	Secondary atom site location: difference Fourier map
*R*[*F*^2^ > 2σ(*F*^2^)] = 0.031	Hydrogen site location: inferred from neighbouring sites
*wR*(*F*^2^) = 0.080	All H-atom parameters refined
*S* = 1.05	*w* = 1/[σ^2^(*F*_o_^2^) + (0.0349*P*)^2^ + 1.1303*P*] where *P* = (*F*_o_^2^ + 2*F*_c_^2^)/3
2783 reflections	(Δ/σ)_max_ = 0.001
205 parameters	Δρ_max_ = 0.27 e Å^−^^3^
0 restraints	Δρ_min_ = −0.20 e Å^−^^3^

### Special details

**Table:**

Geometry. All e.s.d.'s (except the e.s.d. in the dihedral angle between two l.s. planes) are estimated using the full covariance matrix. The cell e.s.d.'s are taken into account individually in the estimation of e.s.d.'s in distances, angles and torsion angles; correlations between e.s.d.'s in cell parameters are only used when they are defined by crystal symmetry. An approximate (isotropic) treatment of cell e.s.d.'s is used for estimating e.s.d.'s involving l.s. planes.
Refinement. Refinement of *F*^2^ against ALL reflections. The weighted *R*-factor *wR* and goodness of fit *S* are based on *F*^2^, conventional *R*-factors *R* are based on *F*, with *F* set to zero for negative *F*^2^. The threshold expression of *F*^2^ > σ(*F*^2^) is used only for calculating *R*-factors(gt) *etc*. and is not relevant to the choice of reflections for refinement. *R*-factors based on *F*^2^ are statistically about twice as large as those based on *F*, and *R*- factors based on ALL data will be even larger.

### Fractional atomic coordinates and isotropic or equivalent isotropic displacement parameters (Å^2^)

**Table:**

	*x*	*y*	*z*	*U*_iso_*/*U*_eq_	
S1	0.55474 (3)	0.22917 (4)	−0.026590 (10)	0.02121 (10)	
N1	0.46643 (10)	0.21163 (13)	0.10454 (3)	0.0194 (2)	
N2	0.46052 (10)	0.18678 (14)	0.05748 (4)	0.0197 (2)	
N3	0.62048 (10)	0.37903 (13)	0.05248 (4)	0.0209 (2)	
C1	0.35320 (12)	0.12835 (15)	0.26361 (4)	0.0198 (3)	
C2	0.46266 (13)	0.20581 (18)	0.28079 (5)	0.0238 (3)	
C3	0.47782 (14)	0.23114 (18)	0.32807 (5)	0.0265 (3)	
C4	0.38429 (14)	0.18207 (18)	0.35891 (5)	0.0283 (3)	
C5	0.27620 (14)	0.10384 (19)	0.34236 (5)	0.0292 (3)	
C6	0.26153 (13)	0.07566 (18)	0.29525 (4)	0.0248 (3)	
C7	0.33153 (12)	0.10103 (16)	0.21392 (4)	0.0203 (3)	
C8	0.39960 (12)	0.16857 (16)	0.17955 (4)	0.0203 (3)	
C9	0.38228 (11)	0.13760 (15)	0.13014 (4)	0.0183 (2)	
C10	0.54759 (11)	0.26906 (15)	0.03065 (4)	0.0174 (2)	
C11	0.71582 (13)	0.48111 (18)	0.02979 (5)	0.0252 (3)	
C12	0.27638 (12)	0.03234 (18)	0.11160 (4)	0.0213 (3)	
HN2	0.4267 (16)	0.097 (2)	0.0461 (6)	0.035 (5)*	
HN3	0.6058 (15)	0.390 (2)	0.0823 (6)	0.031 (4)*	
H2	0.5277 (16)	0.240 (2)	0.2603 (6)	0.034 (4)*	
H3	0.5538 (15)	0.286 (2)	0.3389 (6)	0.033 (4)*	
H4	0.3962 (15)	0.201 (2)	0.3915 (6)	0.032 (4)*	
H5	0.2119 (16)	0.071 (2)	0.3634 (6)	0.040 (5)*	
H6	0.1862 (15)	0.020 (2)	0.2843 (5)	0.034 (4)*	
H7	0.2640 (15)	0.026 (2)	0.2072 (5)	0.026 (4)*	
H8	0.4663 (14)	0.245 (2)	0.1856 (6)	0.026 (4)*	
H11A	0.6826 (18)	0.530 (3)	0.0021 (7)	0.059 (6)*	
H11B	0.7862 (19)	0.419 (3)	0.0221 (7)	0.052 (6)*	
H11C	0.738 (2)	0.572 (3)	0.0489 (7)	0.061 (6)*	
H12A	0.2344 (17)	0.088 (2)	0.0863 (6)	0.038 (5)*	
H12B	0.3056 (16)	−0.073 (2)	0.0991 (6)	0.036 (5)*	
H12C	0.2155 (15)	0.005 (2)	0.1358 (6)	0.033 (4)*	

### Atomic displacement parameters (Å^2^)

**Table:**

	*U*^11^	*U*^22^	*U*^33^	*U*^12^	*U*^13^	*U*^23^
S1	0.02483 (17)	0.02489 (17)	0.01390 (16)	−0.00102 (12)	0.00080 (11)	0.00161 (11)
N1	0.0220 (5)	0.0225 (5)	0.0138 (5)	−0.0007 (4)	−0.0006 (4)	0.0000 (4)
N2	0.0226 (5)	0.0224 (5)	0.0140 (5)	−0.0047 (4)	−0.0002 (4)	0.0002 (4)
N3	0.0236 (5)	0.0208 (5)	0.0183 (5)	−0.0042 (4)	0.0058 (4)	−0.0026 (4)
C1	0.0228 (6)	0.0204 (6)	0.0163 (6)	0.0009 (5)	0.0004 (5)	0.0005 (5)
C2	0.0241 (6)	0.0282 (7)	0.0191 (6)	−0.0035 (5)	0.0001 (5)	0.0012 (5)
C3	0.0304 (7)	0.0275 (7)	0.0217 (7)	−0.0049 (6)	−0.0065 (5)	0.0002 (5)
C4	0.0407 (8)	0.0288 (7)	0.0154 (6)	−0.0017 (6)	−0.0031 (5)	0.0007 (5)
C5	0.0336 (8)	0.0356 (8)	0.0185 (6)	−0.0045 (6)	0.0046 (5)	0.0030 (6)
C6	0.0253 (6)	0.0292 (7)	0.0201 (6)	−0.0052 (5)	0.0005 (5)	0.0011 (5)
C7	0.0214 (6)	0.0216 (6)	0.0179 (6)	−0.0013 (5)	−0.0012 (5)	−0.0014 (5)
C8	0.0217 (6)	0.0212 (6)	0.0180 (6)	−0.0009 (5)	−0.0009 (5)	−0.0013 (5)
C9	0.0197 (6)	0.0183 (6)	0.0170 (6)	0.0019 (5)	0.0002 (4)	0.0010 (5)
C10	0.0168 (5)	0.0176 (5)	0.0177 (6)	0.0035 (4)	0.0001 (4)	0.0019 (4)
C11	0.0245 (6)	0.0258 (7)	0.0252 (7)	−0.0062 (5)	0.0086 (5)	−0.0030 (6)
C12	0.0212 (6)	0.0267 (7)	0.0161 (6)	−0.0029 (5)	0.0004 (5)	0.0000 (5)

### Geometric parameters (Å, º)

**Table:**

S1—C10	1.6875 (12)	C4—H4	0.964 (17)
N1—C9	1.2994 (16)	C5—C6	1.3896 (19)
N1—N2	1.3769 (14)	C5—H5	0.950 (18)
N2—C10	1.3708 (15)	C6—H6	0.967 (17)
N2—HN2	0.862 (18)	C7—C8	1.3402 (18)
N3—C10	1.3261 (16)	C7—H7	0.950 (16)
N3—C11	1.4518 (16)	C8—C9	1.4616 (16)
N3—HN3	0.879 (17)	C8—H8	0.948 (16)
C1—C6	1.3980 (18)	C9—C12	1.4981 (17)
C1—C2	1.4029 (18)	C11—H11A	0.96 (2)
C1—C7	1.4716 (17)	C11—H11B	0.92 (2)
C2—C3	1.3917 (18)	C11—H11C	0.94 (2)
C2—H2	0.949 (18)	C12—H12A	0.965 (18)
C3—C4	1.388 (2)	C12—H12B	0.964 (18)
C3—H3	0.965 (17)	C12—H12C	0.977 (17)
C4—C5	1.387 (2)		
			
C9—N1—N2	117.87 (10)	C8—C7—C1	125.58 (12)
C10—N2—N1	117.43 (10)	C8—C7—H7	120.2 (9)
C10—N2—HN2	117.2 (11)	C1—C7—H7	114.3 (9)
N1—N2—HN2	121.0 (11)	C7—C8—C9	126.19 (12)
C10—N3—C11	123.89 (11)	C7—C8—H8	121.4 (10)
C10—N3—HN3	115.2 (11)	C9—C8—H8	112.4 (10)
C11—N3—HN3	120.8 (11)	N1—C9—C8	113.27 (11)
C6—C1—C2	118.21 (12)	N1—C9—C12	124.17 (11)
C6—C1—C7	119.15 (11)	C8—C9—C12	122.55 (11)
C2—C1—C7	122.64 (11)	N3—C10—N2	115.86 (11)
C3—C2—C1	120.46 (12)	N3—C10—S1	124.41 (9)
C3—C2—H2	119.3 (10)	N2—C10—S1	119.73 (9)
C1—C2—H2	120.3 (10)	N3—C11—H11A	110.6 (12)
C4—C3—C2	120.56 (13)	N3—C11—H11B	111.8 (13)
C4—C3—H3	120.8 (10)	H11A—C11—H11B	108.3 (17)
C2—C3—H3	118.7 (10)	N3—C11—H11C	109.8 (13)
C5—C4—C3	119.49 (12)	H11A—C11—H11C	105.7 (18)
C5—C4—H4	121.0 (10)	H11B—C11—H11C	110.4 (18)
C3—C4—H4	119.5 (10)	C9—C12—H12A	111.0 (10)
C4—C5—C6	120.18 (13)	C9—C12—H12B	112.4 (10)
C4—C5—H5	119.6 (11)	H12A—C12—H12B	105.3 (14)
C6—C5—H5	120.2 (11)	C9—C12—H12C	111.2 (10)
C5—C6—C1	121.07 (13)	H12A—C12—H12C	110.1 (14)
C5—C6—H6	119.2 (10)	H12B—C12—H12C	106.6 (14)
C1—C6—H6	119.7 (10)		
			
N1—N1—N2—C10	0.00 (9)	N2—N1—C9—N1	0 (100)
C9—N1—N2—C10	178.27 (11)	N1—N1—C9—C8	0.00 (7)
C9—N1—N2—N1	0 (100)	N2—N1—C9—C8	178.31 (10)
C6—C1—C2—C3	−0.9 (2)	N1—N1—C9—C12	0.000 (19)
C7—C1—C2—C3	178.99 (13)	N2—N1—C9—C12	−2.44 (18)
C1—C2—C3—C4	−0.8 (2)	C7—C8—C9—N1	−176.06 (12)
C2—C3—C4—C5	1.4 (2)	C7—C8—C9—N1	−176.06 (12)
C3—C4—C5—C6	−0.3 (2)	C7—C8—C9—C12	4.7 (2)
C4—C5—C6—C1	−1.4 (2)	C11—N3—C10—N2	−178.42 (12)
C2—C1—C6—C5	2.0 (2)	C11—N3—C10—S1	0.53 (18)
C7—C1—C6—C5	−177.89 (13)	N1—N2—C10—N3	−7.04 (16)
C6—C1—C7—C8	168.26 (13)	N1—N2—C10—N3	−7.04 (16)
C2—C1—C7—C8	−11.7 (2)	N1—N2—C10—S1	173.96 (9)
C1—C7—C8—C9	177.50 (12)	N1—N2—C10—S1	173.96 (9)

### Hydrogen-bond geometry (Å, º)

**Table:**

*D*—H···*A*	*D*—H	H···*A*	*D*···*A*	*D*—H···*A*
N3—H*N*3···N1	0.879 (17)	2.143 (16)	2.5877 (15)	110.7 (13)
N2—H*N*2···S1^i^	0.862 (18)	2.663 (18)	3.4296 (12)	148.7 (15)

Symmetry code: (i) −*x*+1, −*y*, −*z*.
